# HIV trend among Iranian prisoners in 1990s and 2000s; analysis of aggregated data from HIV sentinel sero-surveys

**DOI:** 10.1186/1477-7517-10-32

**Published:** 2013-11-20

**Authors:** Ali-Akbar Haghdoost, Ali Mirzazadeh, Mostafa Shokoohi, Abbas Sedaghat, Mohammad Mahdi Gouya

**Affiliations:** 1 Regional Knowledge Hub, and WHO Collaborating Centre for HIV Surveillance, Institute for Futures Studies in Health, Kerman University of Medical Sciences, Kerman, Iran; 2 Research Center for Modeling in Health, Institute for Futures Studies in Health, Kerman University of Medical Sciences, Kerman, Iran; 3 Institute for Health Policy Studies, University of California, San Francisco, CA, USA; 4 Ministry of Health & Medical Education, CDC, Tehran, Iran

**Keywords:** Sentinel sero-survey, Prevalence, HIV testing, Prisoners, Iran

## Abstract

**Background:**

Using the aggregated data of all sentinel sero-surveys (1991 to 2007), this study aimed to report the HIV trend among Iranian prisoners.

**Method:**

Overall, we analysed the aggregated data from 397 HIV sero-surveys conducted in 72 prisons (included 155,771 prisoners) from 1991 through 2007.

**Results:**

The overall HIV prevalence was 2.8% (95% CI: 1.8%-4.3%). In 1998, HIV prevalence dramatically increased to 4.5% (95% CI: 1.1%-16.8%), which later became stable at level of 2.8%. Prisons were so heterogeneous regarding HIV prevalence (0% to 13.2%).

**Conclusion:**

Since the outbreak, the ministry of health has acknowledged prisoners as one of the high-risk groups for HIV, increased the number of sentinel surveys and on-site harm reduction services to better monitor and response to the HIV epidemic. The downward trend of HIV prevalence after 2005 suggests the effectiveness of such interventions which need to be continued.

## Background

Iran, with very strict rules against drug trafficking, has been used as one of the main transient routes for drugs and opium trafficking from Afghanistan to other countries. Consequently, this has made opium and other drugs easily available for Iranians [1,2]. This put more people at risk of drug addiction and drug trafficking related crimes, and so more related incarceration [3,4].

Given the fact that HIV epidemic in Iran is driven by injecting drug users, prisoners could be at higher risk of HIV acquisition and further transmission [5-7]. Prisoners are considered as one of the high-risk groups for HIV in many countries [6,8]. High prevalence of HIV among prisoners in compare to general population was reported in USA (ratio 6:1), France (ratio 10:1) [9].

In Iran, HIV outbreak in prisons was first observed in mid-1990s [4]. The outbreak in Kermanshah prisons grasped national attention, when the first index HIV cases were reported in 1995, followed by 58 confirmed cases in 1996. Later, the number of HIV infected prisoners increased to 407 cases in 1997–8. This was reported as the first HIV outbreak inside prison in Iran [4,10].

Health authorities used the advantage of these outbreaks to advocate for comprehensive control measures in- and out-side prisons. Since 2003, the harm reduction programmes implemented and expanded by increasing the number of triangular clinics, setting up a free and voluntary HIV consulting and testing, establishing the methadone maintenance therapy, spreading the bleach and disposal razors, distributing free condoms, starting the needle and syringe exchange programs, educating prisoners and their families, and running the psychotherapy meetings widely, particularly among injecting drug users, both in- and out-side prisons [11-15].

Meanwhile, to monitor the trend of HIV prevalence within prisons, HIV sentinel sero-surveys have been implemented widely among prisons, in particular after 1998 outbreak. Such data are available since 1991, but not yet aggregated, analyzed and reported. In this paper, using the existing HIV sentinel surveillance data, we explore the HIV trend among prisoners since early 1990s to monitor the response to prisons’ HIV epidemics in Iran.

## Methods

For this analysis, all 397 HIV sentinel sero-surveys were included which implemented in 72 prisons since 1991. The venues blood sample was obtained from every recruited prisoner and was tested for anti-HIV antibody using the enzyme-linked immunosorbent assay (ELISA) method. It was considered as positive if reconfirmed by a second ELISA test. The surveys were designed and implemented jointly by Prisons Organization and Center for Disease Control (CDC). The data of the present study was collected from 1991 through 2007.

To produce the large database, first the pooled results of every survey to produce individual records having HIV status, year and prison name were expanded. Later, all separated expanded databases were merged into one database for analysis. The final large database was consisted of 155,771 records as prisoners. Written informed consent was obtained from the patient for the publication of this report. The protocols of these surveys and providing the information of this study have been approved by the Ministry of Health (MOH), Iran and ethical committee of Kerman University of Medical Sciences.

Then, the HIV prevalence was calculated overall and by year and province. In this analysis, prisons were considered as the sampling units (clusters). Intra cluster coefficient (ICC) was reported as a measure for intra-prison correlation coefficient of HIV. Finally, using Geographical Information System (GIS) the HIV spatial distribution in prisons was illustrated at the level of provinces.

## Results

In total, 155,771 prisoners were recruited in the sentinel surveys, with the average sample size of 392 (range 10 to 2,200). A few surveys were implemented before 1998 HIV outbreak. Since then, the number of surveys has surged dramatically; so that in recent years more than 50 surveys have been annually carried out in prisons.

**Figure 1:**
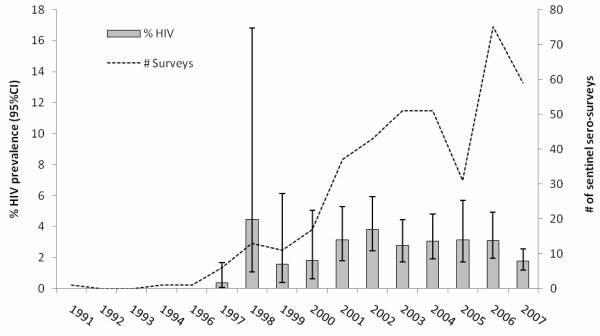
HIV prevalence among prisoners and number of sentinel sero-surveys implemented in prisons between 1991 and 2007 in Iran.

Overall HIV prevalence was 2.8% (95% CI: 1.8%-4.3%). While the HIV was detected at 0% to 0.4% in prisons before 1998, due to the HIV outbreak in 1998, the HIV prevalence reached to the maximum of 4.5% (95% CI 1.1%-16.8%). After 1998, HIV prevalence was at 2.8% on average, ranged from 1.5% to 3.8% (Figure 1).

**Figure 2:**
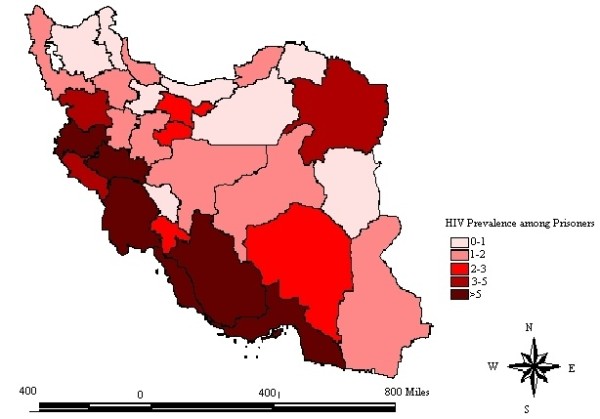
The spatial distribution of HIV prevalence among prisoners between 1991 and 2007.

HIV prevalence varied from 0% to 13.2% in different prisons. Prisoners located in provinces in west and south part of Iran had a higher prevalence of HIV (range 3.4% - 13.2%) in comparison with other provinces (Figure 2).

Using Poisson regression model, a significant intra-prison correlation was observed regarding the HIV prevalence; (Intra Cluster Coefficient: Overall ICC =0.09; for surveys between 2000 and 2007: ICC = 0.07; for survey between 2000 and 2007: ICC = 0.06).

## Discussion

The overall HIV prevalence was 2.8%, which is higher than that of the general population (around 0.1%) [5]. The results demonstrated that the prevalence of HIV in recent years is more or less constant at the level of 3%, if not decreasing. However, due to limited surveys conducted prior to 1998, it is difficult to generalize our finding to early 1990s.

Farnia et al. in their survey among Iranian prisoners in 2005 estimated the overall HIV prevalence as the level of 3% (ranged from 0.2% in Khorasan province to 13.5% in Kermanshah province) [16]. This pattern is very similar to what was found in our study.

In recent bio-behavioural study of 5530 prisoners from 27 prisons in 2009 in Iran [7], the HIV prevalence was reported at 2.1% (95% CI: 1.2 - 3.6). This was comparable to the findings of the present study. Regarding the prisoners risk profile, only one-fifth (20.5%) had comprehensive knowledge about HIV, and one-fourth (24.7%) had used condom in their last sexual contact inside prison. In addition, the overall history of drug injection was reported by 16.5% of all prisoners and there has been a strong association between ever drug injection and being HIV positive (8.1% in ever injected vs. 0.9% in never injected). In Ohaio prison [17], approximately 12.1% of inmates reported sexual contact during incarceration, that in around 85% of the time it was with multiple partners. Only 15.4% used condom during sexual relationship. These behavioural findings indicate that prisoners are still at risk of HIV acquisition and transmission; therefore, it should be monitored by both sentinel sero-surveys and behavioural surveillance surveys. Specific interventions such as harm reduction for both sexual and injection related behaviours and providing sufficient and convenient treatment services are needed to decrease HIV transmission risk among prisoners [18-22].

Since 1998, any drug user (either injecting or non-injecting) as far as they only identified as a drug user, were considered as patient not as a criminal. So, they were not arrested and be put in jail and prison, but referred to drug treatment and rehabilitation centers. This was a remarkable amendment in the anti-narcotic law and judiciary system. The sharp decrease in HIV prevalence inside the prisons in 1999 and 2000 could be partly explained by reduction in the number of prisoners at risk for HIV, like injecting drug users, inside the prison as the effect of the implementation of such policy.

In 2000, first triangular clinic was established inside Kermanshah prison and later expanded to other prisons [10]. They provided confidential HIV counseling and testing services as well as anti-retroviral treatment to eligible HIV positive patients (based on national HIV treatment guideline). Since 2003, harm reduction services/programs with the focus on needle/syringe exchange programs and methadone maintenance therapy (expanded to all 30 provinces included 142 prisons in 2010) became available to prisoners [13,23]. Such ongoing harm reduction intervention package for prisoners [15,24,25] can explain the downward trend of HIV prevalence after 2005 indicates the effectiveness of such interventions and continuing these programs are needed. This needs further investigation as the downward trend could be due to higher mortality.

As prisoners might acquire the HIV infection either outside or inside the prison, sero-surveys of prisoners at the point of entry to prison would help to better understand the source of HIV infections among prisoners. This will also lead health authorities to plan and implement more effective interventions.

We should acknowledge the limitations of our study. At the very beginning of the HIV epidemic in prisons, the identified HIV positive cases have been isolated from the rest of the prisoners. We do not know the exact starting date of the isolation strategy and we cannot distinguish which of the sero-surveys might have been affected by it. The effect of such isolation would be an underestimate of HIV prevalence and an unrealistic downward trend. Since the isolation strategy was implemented by about 2000 and discontinued, the sharp decrease between 1998 and 1999–2000 could be partly explained by it, but it would not affect the observed trend in the recent decade.

Basically, sentinel surveys only monitor HIV prevalence not related behaviours. Therefore, we were not able to report the HIV epidemics by individual characteristics and behavioural profile. In addition, to get a more accurate picture for the HIV trend in prisons and to plan for more effective preventive interventions, repeated sero-surveys within every prison is needed. The focus should be on prisons with either upward trend or stable but high level of HIV over time.

## Conclusion

The overall HIV prevalence among prisoners is 2.8%, and this rate has been almost constant during recent years in Iran. Since the outbreak, the ministry of health has acknowledged prisoners as one of the high-risk groups for HIV, increased the number of sentinel surveys and on-site harm reduction services to better monitor and response to the HIV epidemic. The downward trend of HIV prevalence after 2005 indicates the effectiveness of such interventions which need to be continued.

## Competing interest

All authors declare that they have no competing interest.

## Authors contribution

A-AH participated in the design of the study, analysis and interpretation of the findings; drafting the article. AM and MS contributed to the concept and design; monitoring of data collection and quality, analysis and interpretation of data; with lead roles in carrying out literature review, drafting the article AS and MMG contributed to the design, interpretation of findings. All authors read and approved the final manuscript.

## References

[B1] Noohi S Azar M Heshmatzade Behzadi A Sedaghati M Akbari Panahi S Dehghan N A Comparative Study of Characteristics and Risky Behaviors among the Iranian Opium and Opium Dross Addicts J Addict Med 2011 5 74 78 10.1097/ADM.0b013e3181db69ef 21769050

[B2] Gibson A Degenhardt L Topp L Day C Hall W Dietze P McKetin R Global and Australian heroin markets 2003 Sydney: National Drug and Alcohol Research Centre, University of New South Wales

[B3] Iran Prison Organization An overview on HIV/AIDS in prisons of The Islamic Republic of Iran by Health and Treatment Headquarter 2006 Tehran: Iran Prison Organization

[B4] Zamani S Farnia M Torknejad A Abbasi Alaei B Gholizadeh M Kasraee F Patterns of Drug Use and HIV-Related Risk Behaviors among Incarcerated People in a Prison in Iran J Urban Health 2010 87 4 603 616 10.1007/s11524-010-9450-8 20390391 PMC2900562

[B5] National AIDS Committee Secretariat, Ministry of Health and Medical Education. Islamic Republic of Iran Progress Report on Monitoring of United Nations General Assembly Special Session (UNGASS) on HIV and AIDS 2008 The full text available at: http://www.unaids.org/en/dataanalysis/knowyourresponse/countryprogressreports/2012countries/IRIran%20AIDS%20Progress%20Report%202012%20English%20final1_1.pdf

[B6] Zamani S Kihara M Gouya MM High prevalence of HIV infection associated with incarceration among community-based injecting drug users in Tehran, Iran J Acquir Immune Defic Syndr 2006 42 3 342 346 10.1097/01.qai.0000219785.81163.67 16639351

[B7] Navadeh S Mirzazadeh A Gouya MM Farnia M Alasvand R Haghdoost AA HIV prevalence and related risk behaviors among prisoners in Iran: Results of the national bio-behavioral survey, 2009 Sex Transm Infect 2013 0 1 4 doi:10.1136/sextrans-2013-051295 10.1136/sextrans-2013-051295 PMC3841726 23986417

[B8] Dolan K Kite B Black E Aceijas C Stimson GV HIV in prison in low-income and middle-income countries Lancet Infect Dis 2007 7 1 32 41 10.1016/S1473-3099(06)70685-5 17182342

[B9] UN Office on Drug and Crime, UNAIDS and World Bank HIV and Prisons in Sub-Saharan Africa: Opportunities for action 2007 Vienna: UN Office on Drug and Crime available at: http://www.unodc.org/documents/hiv-aids/Africa%20HIV_Prison_Paper_Oct-23-07-en.pdf

[B10] World Health Organization Best practice in HIV/AIDS prevention and care for injecting drug abusers: the Triangular Clinic in Kermanshah, Islamic Republic of Iran 2004 WHO, Regional Office for the Eastern Mediterranean Cairo

[B11] Vazirian M Nassirimanesh B Zamani S Ono-Kihara M Kihara M Ravari SM Needle and syringe sharing practices of injecting drug users participating in an outreach HIV prevention program in Tehran, Iran: A cross-sectional study Harm Reduction J 2005 2 19 10.1186/1477-7517-2-19 PMC1266391 16212655

[B12] Razzaghi EM Movaghar AR Green TC Khoshnood K Profiles of risk: A qualitative study of injecting drug users in Tehran, Iran Harm Reduction J 2006 3 12 10.1186/1477-7517-3-12 PMC1431517 16545137

[B13] Razzaghi E Nassirimanesh B Afshar P Ohiri K Claeson M Power R HIV/AIDS harm reduction in Iran Lancet 2006 368 9534 434 435 10.1016/S0140-6736(06)69132-0 16890814

[B14] Malekinejad M Vazirian M Transition to injection amongst opioid users in Iran: Implications for harm reduction Int J Drug Policy 2012 23 333 337 10.1016/j.drugpo.2011.09.001 21996166

[B15] Eshrati B Asl RT Dell CA Preventing HIV transmission among Iranian prisoners: initial support for providing education on the benefits of harm reduction practices Harm Reduction J 2008 5 21 10.1186/1477-7517-5-21 PMC2443130 18541032

[B16] Farnia M Ebrahimi B Shams A Zamani S Scaling up methadone maintenance treatment for opioid-dependent prisoners in Iran Int J Drug Policy 2010 21 5 422 424 10.1016/j.drugpo.2010.03.008 20413287

[B17] Sieck CJ Dembe AE Results of a Pilot Study of Pre-release STD Testing and Inmates’ Risk Behaviors in an Ohio Prison J Urban Health 2011 88 4 690 699 10.1007/s11524-011-9565-6 21448579 PMC3157500

[B18] Rosen DL Schoenbach VJ Wohl DA White BL Stewart PW Golin CE Characteristics and Behaviors Associated With HIV Infection among Inmates in the North Carolina Prison System Am J Public Health 2009 99 6 1123 1130 10.2105/AJPH.2007.133389 19372527 PMC2679772

[B19] Burchell AN Calzavara LM Myers T Schlossberg J Millson M Escobar M Voluntary HIV Testing Among Inmates: Sociodemographic, Behavioral Risk, and Attitudinal Correlates JAIDS 2003 32 5 534 541 10.1097/00126334-200304150-00011 12679706

[B20] Alistar S Owens D Brandeau M Effectiveness and cost effectiveness of expanding harm reduction and antiretroviral therapy in a mixed HIV epidemic: a modeling analysis for Ukraine PLoS Med 2011 8 3 E1000423 10.1371/journal.pmed.1000423 21390264 PMC3046988

[B21] Ohiri K Claeson M Rassaghi E HIV/AIDS Prevention among Injection Drug Users: Learning from Harm Reduction in Iran 2006 Iran: HIV Prevention Consultation

[B22] Hasnain M Cultural Approach to HIV/AIDS Harm Reduction in Muslim Countries Harm Reduction J 2005 27 2 23 10.1186/1477-7517-2-23 PMC1298319 16253145

[B23] Eshrati B Asl RT Dell CA Preventing HIV transmission among Iranian prisoners: initial support for providing education on the bene fits of harm reduction practices Harm Reduct J 2008 5 21 10.1186/1477-7517-5-21 18541032 PMC2443130

[B24] Jenkins C Rahman H Saidel T Measuring the impact of needle Exchange Programs among injecting drug users through the National Behavioral Surveillance in Bangladesh AIDS Educ Prev 2001 13 5 452 461 10.1521/aeap.13.5.452.24141 11718444

[B25] Zamani S Farnia M Tavakoli S A qualitative inquiry into methadone maintenance treatment for opioid-dependent prisoners in Tehran, Iran Int J Drug policy 2010 21 3 167 172 10.1016/j.drugpo.2009.03.001 19395250

